# Near-Infrared Light Exposure Triggers ROS to Downregulate Inflammatory Cytokines Induced by SARS-CoV-2 Spike Protein in Human Cell Culture

**DOI:** 10.3390/antiox12101824

**Published:** 2023-10-02

**Authors:** Blanche Aguida, Marie-Marthe Chabi, Soria Baouz, Rhys Mould, Jimmy D. Bell, Marootpong Pooam, Sebastien André, Dominique Archambault, Margaret Ahmad, Nathalie Jourdan

**Affiliations:** 1 UMR8256, CNRS, IBPS, Sorbonne University, 75005 Paris, France; blanche.aguida@sorbonne-universite.fr (B.A.);; 2 Research Centre for Optimal Health, University of Westminster, London W1W 6UW, UKj.bell@westminster.ac.uk (J.D.B.); 3 Department of Biology, Faculty of Science, Naresuan University, Phitsanulok 65000, Thailand; marootpongp@nu.ac.th; 4 Nutrition and Obesities: Systemic Approaches, NutriOmics, Research Unit, Sorbonne University, INSERM, 75013 Paris, France; 5 Laboratoire CHArt, University of Paris 8-Vincennes-Saint-Denis, 93526 Saint-Denis, France; 6 Department of Biology, Xavier University, 3800 Victory Parkway, Cincinnati, OH 45207, USA

**Keywords:** SARS-CoV-2, near-infrared light exposure, TLR4 inflammatory signaling pathway, reactive oxygen species (ROS), human primary alveolar cells, human macrophage cell line, mitochondria, antioxidant genes, photobiomodulation therapy

## Abstract

The leading cause of mortality from SARS-CoV-2 is an exaggerated host immune response, triggering cytokine storms, multiple organ failure and death. Current drug- and vaccine-based therapies are of limited efficacy against novel viral variants. Infrared therapy is a non-invasive and safe method that has proven effective against inflammatory conditions for over 100 years. However, its mechanism of action is poorly understood and has not received widespread acceptance. We herein investigate whether near-infrared (NIR) light exposure in human primary alveolar and macrophage cells could downregulate inflammatory cytokines triggered by the SARS-CoV-2 spike (S) protein or lipopolysaccharide (LPS), and via what underlying mechanism. Our results showed a dramatic reduction in pro-inflammatory cytokines within days of NIR light treatment, while anti-inflammatory cytokines were upregulated. Mechanistically, NIR light stimulated mitochondrial metabolism, induced transient bursts in reactive oxygen species (ROS) and activated antioxidant gene transcription. These, in turn, downregulated ROS and inflammatory cytokines. A causal relationship was shown between the induction of cellular ROS by NIR light exposure and the downregulation of inflammatory cytokines triggered by SARS-CoV-2 S. If confirmed by clinical trials, this method would provide an immediate defense against novel SARS-CoV-2 variants and other inflammatory infectious diseases.

## 1. Introduction

Severe acute respiratory syndrome coronavirus 2 (SARS-CoV-2) is a highly pathogenic *β-coronavirus* that recently emerged in humans [1] and led to the global pandemic of coronavirus disease-2019 (COVID-19). Mortality ensues from an excessive, uncontrolled inflammatory response, the so-called cytokine storm, leading to acute lung injury (ALI) and acute respiratory distress syndrome (ARDS). This may be followed by multi-organ failure and death, particularly in the elderly and in individuals with co-morbidity risk factors [2,3]. There are currently no effective antiviral treatments and no effective treatments against late-stage COVID-19, and existing vaccines are of limited effectiveness due to multiple mutations harbored by newly emerging SARS-CoV-2 variants [4,5]. The same exacerbated immune responses, characterized by cytokine storms, ARDS and multi-organ failure are also characteristic of two other highly pathogenic *β-coronaviruses* that emerged in 2002 and 2013: severe acute respiratory syndrome coronavirus (SARS-CoV) [6] and Middle East respiratory syndrome coronavirus (MERS-CoV) [7], respectively. Therefore, SARS-CoV-2 is the third *β-coronavirus* to emerge since the beginning of the 21st century, indicating that *β-coronavirus* has high outbreak and pandemic potential and continues to pose a significant threat to public health.

Patients with severe COVID-19 start to develop hyperinflammation in the lower respiratory tract. The key cell types mediating this dysregulated innate immunity are type II alveolar epithelial cells together with resident and recruited macrophages [8,9]. Past studies have reported that the hyperinflammatory pathology triggered in COVID-19 patients is associated with an upregulation of Toll-like receptor (TLR) 4 in peripheral blood mononuclear cells (PBMCs) in severe COVID-19 patients, compared to healthy controls [10]. TLRs are transmembrane proteins expressed in epithelial and immune cells, and their normal function is to induce innate immune responses by recognizing PAMPs (pathogen-associated molecular patterns) or DAMPs (danger-associated molecular patterns) circulating in the bloodstream. Binding to PAMPs or DAMPs initiates the Nuclear Factor κB (NF-κB) activation pathway, followed by the transcription and secretion of pro-inflammatory cytokines [11]. Of the 10 members of the TLR family in human cells, TLR4 is the main receptor for the recognition of Gram-negative bacterial lipopolysaccharide (LPS) [12]. The ensuing TLR4/NF-κB signaling is an important host defense mechanism to eliminate bacterial infection. However, excessive TLR4/NF-κB activation may occur in response to high LPS levels and result in fatal septic shock [13]. Similar severe overactivation of the TLR4/NF-κB pathway has been implicated in critical COVID-19 patients [14]. The SARS-CoV-2 spike protein (S1 subunit) is shown to interact with TLR4 [14,15,16], and to trigger a dysregulated increase in inflammatory cytokines comparable to that induced by LPS in human monocyte cell lines [14,16].

Recently, the use of photobiomodulation therapy (PBM) has been suggested as a possible treatment for hyperinflammatory cytokine storms induced in COVID-19 patients [17,18]. PBM, which involves the medical use of low-intensity light, has been used for over 100 years as a non-invasive, safe and effective treatment in which diseased tissue is exposed to a few minutes of long-wavelength red or infrared light, repeated once or twice daily. PBM has been proven clinically effective in osteoarthritis [19]; bone [20] and wound [21] healing; and for other conditions with underlying inflammation such as thyroiditis [22], psoriasis and chronic pain [23]. In keeping with its anti-inflammatory effects, PBM treatment has been shown to suppress pro-inflammatory cytokine secretion and deactivate the inflammasome in a variety of cell culture and animal disease models [19,23,24,25]. The advantages of PBM over other forms of therapy are the absence of side effects and the possibility to specifically target the diseased tissue. Significantly, PBM has also been reported to be effective against ALI and other pulmonary inflammatory conditions in animal models [24,26,27], consistent with the benefits of treatment for COVID-19-associated pathology. Indeed, several case reports and small-scale clinical studies were performed at the height of the recent pandemic using PBM to treat hyperinflammatory cytokine storms in hospitalized COVID-19 patients, with encouraging preliminary results [28,29,30,31]. Finally, using a human TLR4 reporter model cell line, prior work by our own team has shown that NIR light exposure can be effective in treating TLR4-dependant inflammation of the type induced by SARS-CoV-2, reducing secretion of the inflammatory cytokine IL-6 by almost 80% within only two days of PBM treatment [32,33].

However, in spite of these promising indications, PBM therapy in general has yet to reach mainstream acceptance. The mechanism of action is not understood and there is a marked lack of consistency in PBM exposure protocols and results [23]. Currently, there is a consensus that red/infrared light is absorbed by mitochondrial cytochrome C oxidase, leading to an increase in mitochondrial function. The resulting increases in ATP, cyclic AMP, calcium flux, nitric oxide (NO) and the activation of hypoxia-inducible factor 1 alpha (HIF-1α) and other transcription factors have been variously suggested as mediators of the therapeutic effects of PBM [23,34]. Another suggestion has been that cellular ROS induced by PBM exposure may modulate the immune response and explain the anti-inflammatory effects of PBM [35,36]. However, there is as yet no direct evidence for these hypotheses, and current therapeutic protocols are largely a matter of trial and error.

Here, we address two of the major outstanding issues concerning the effectiveness of PBM therapy for treating hyperinflammation induced by COVID-19. Firstly, we verify that PBM is effective in downregulating the TLR4-dependent inflammatory response in the two cell types most directly impacted by COVID 19: primary human alveolar type II cells (HAECs) and a human macrophage cell line (THP-1).

Secondly, we determine that ROS plays a central role in the underlying mechanism involved in these anti-inflammatory effects. We show that NIR light induces a transient increase in cellular H_2_O_2_, and that the exogenous application of H_2_O_2_ to cell cultures achieves identical downregulation of the inflammatory pathway. Therefore, the modulation of ROS by NIR light exposure is necessary and sufficient to explain these anti-inflammatory effects. Broader implications for the eventual treatment of inflammatory infectious diseases are discussed.

## 2. Materials and Methods

### 2.1. Cell Lines and Cultures

Human type II alveolar epithelial cells (HAECs) isolated from human normal lung tissue were purchased from AcceGen (AcceGen Biotech, Fairfield, NJ, USA) and cultured in an HAEC medium kit according to the manufacturer’s instructions. The human embryonic kidney HEK293 cell line was kindly provided by Professor B. Friguet (Sorbonne Université, Paris, France) and grown in Minimum Essential Medium Eagle (MEM) (Merck, Darmstadt, Germany), supplemented with L-Alanyl-L-Glutamine (0.4 g/L) and 10% heat-inactivated fetal calf serum (FCS) (Gibco, Dublin, Ireland). HEK-BLUE-hTLR4 stably expressing human TLR4 and an inducible SEAP (secreted embryonic alkaline phosphatase) reporter gene was purchased from InvivoGen (San Diego, CA, USA) and grown in Dulbecco’s Modified Eagle Medium (DMEM)—high glucose (Merck), supplemented with 10% heat-inactivated FCS and antibiotic mixture (HEK-Blue^TM^ Selection) for the persistent expression of transgenes. The human monocytic leukemia cell line (THP-1) was kindly provided by Dr S. André (Sorbonne Université, Paris, France) and maintained in RPMI 1640 (Gibco) supplemented with 10% heat-inactivated FBS, 100 units/mL penicillin and 100 μg/mL streptomycin. THP-1 was grown to a density of 500,000 cells/mL and used for experiments between passage numbers 5 and 10. All cells were cultured under a humidified atmosphere in a 95% air–5% CO2 incubator at 37 °C.

### 2.2. Expression and Purification of Recombinant SARS-CoV-2 Spike Protein

A pCMV plasmid encoding the ancestral strain Wuhan-Hu-1 SARS-CoV-2 Spike (S) Glycoprotein Ectodomain, NR-52421, was contributed by David Veesler for distribution through BEI Resources, NIAID, NIH. NR-52421 was designed by codon-optimizing the S glycoprotein ectodomain (residues 14 to 1211) for mammalian expression, fused to an *N*-terminal mu-phosphatase signal sequence and C-terminal trimerizing foldon domain and octa-histidine tag [37]. The SARS-CoV-2 Spike glycoprotein ectodomain was produced via the transfection of NR-52421 in the human embryonic kidney HEK293 cell line, purified via nickel affinity (Ni-NTA agarose) chromatography and vialed in phosphate-buffered saline (PBS), pH 7.4.

### 2.3. TLR4/NF-κB Inflammatory Signaling Pathway Activation and Near-Infrared Exposure

HEK-Blue™-hTLR4 cells were seeded at a density of 1.3 × 10^7^ cells in 22.1 cm^2^ plates and stimulated with 100 ng/mL of LPS from Escherichia coli 0111:B4 (LPS-B4 Ultrapure; Sigma Aldrich, Saint-Quentin-Fallavier, France) to induce the TLR4/NF-κB inflammatory pathway. HAECs were seeded at a density of 5 × 10^5^ cells in 22.1 cm^2^ plates and stimulated with 1 μg/mL of LPS. Human THP-1 cells were seeded at a density of 10^7^ cells in 22.1 cm^2^ plates and incubated with 50 nM of PMA (Merck) for 48 h for differentiation to a macrophage phenotype. After 48 h, the PMA-containing medium was replaced with fresh medium and the cells were allowed to rest in culture for 24 h before stimulation with 100 ng/mL LPS (100 nM) or 10 nM SARS-CoV-2 Spike Glycoprotein Ectodomain. The day after TLR4/NF-κB activation, all cells were submitted to near-infrared exposure using a Cool–IR 730 nm Bulb obtained from Synlyte, 91300 Massy Palaiseau, FR (www.synlyte.com, accessed on 28 September 2023), at an intensity of 6 W/m^2^ at the position of the cells. The infrared treatment was applied for 10 min every 12 h over a total time of 48 h (for HEK-Blue™-hTLR4 cells and HAECs) or 96 h (for THP-1). To maintain TLR4/NF-κB activation during the whole experiment, an additional boost of LPS or S was performed at days 2 and 3 (for HEK-Blue™-hTLR4 and HAECs) or days 6, 7 and 8 (for THP-1) of the experiment. The control condition was performed in an identical manner and the sample cultured in darkness without infrared illumination.

### 2.4. Light Source and Exposure Conditions

Near-infrared (NIR) light exposure was achieved using a 730 nm Synlyte PulseIR Custom LED lamp (https://synlyte.com/product/synlyte-tm-pulseir-custom-led-lamp-750-e/, accessed on 28 September 2023). The optimum light exposure conditions have been previously defined [32,33]. Briefly, the lamp was positioned at a distance of 20 cm above the cells and adjusted at a fluence of 6 W/m^2^ at the level of the cells for a total continuous light exposure time of 10 min.

### 2.5. Measure of Real-Time CELL Temperature Fluctuation during NIR Exposure

Real-time cell temperature fluctuation monitoring was achieved using a commercially available waterproof DS18B20 digital temperature sensor connected to custom-built electronic device in charge of recording temperature every second. The device is based on a Lolin D32 prototype board from Wemos, based on the Espressif ESP32-WROOM-32 micro-controller, including an original code implemented in Micro Python. The code includes a time correction algorithm based on processing execution time, allowing us to ensure that the temperature is read every 1000 ms ± 2 ms on average. Inside the incubator, the temperature sensor was immersed in the cell culture medium and temperature recording began 20 min before NIR exposure, during the 10 min exposure, and continued for 20 min after the end of exposure. The data were stored locally in an SD card within the device during the experiment in CSV format.

### 2.6. Quantitative RT-PCR Analysis of Inflammatory Cytokines and Antioxidant ENZYME Genes 

Three hours after the last boost of the NIR light exposure protocol, total RNA was extracted from HAECs or THP-1 cells using a Total RNA Miniprep Kit (New England Biolabs, Evry, France), according to the manufacturer’s instructions. cDNA was synthesized from 1 µg total RNA using a ProtoScript^®^ II First Strand cDNA Synthesis Kit (New England Biolabs, Evry, France). Quantitative RT-PCR was performed using Luna qPCR master mix (New England Biolabs, Evry, France). The specific primers are listed in Supplementary Table S1. Mastercycler^®^ RealPlex2 (Eppendorf, Evry-Courcouronnes, France) was used to perform amplification with the following thermal cycling conditions: denaturation at 95 °C for 1 min followed by 40 cycles of denaturation at 95 °C for 15 s, and annealing and elongation at 60 °C for 45 s. A dissociation curve for each well was performed by running the following program: 95 °C for 15 s, 60 °C for 15 s and 60 to 95 °C at 2 °C/min. The obtained Ct (cycle threshold) values of the target genes were normalized to the NAPDH housekeeping gene, and the 2^−ΔΔCT^ method was used to calculate fold changes [38]. Three biological replicates were performed for each gene (*n* = 3).

### 2.7. Detection of IL-6 Secretion via ELISA

Six hours after the last boost of the NIR light exposure protocol, HAECs and THP-1 cell supernatants were harvested, and IL-6 secreted into the medium during the final 6 h of the experiment was measured using a Human IL-6 DuoSet ELISA kit, according to the manufacturers’ instructions (R&D SYSTEMS a Bio-Techne brand, San Jose, CA 95134, USA).

### 2.8. Measurement of Mitochondrial Calcium (Ca^2+^)

HEK-Blue™-hTLR4 cells were seeded on black 96-well clear-bottom plates at a seeding density of 1.25 × 10^4^ cells per well, stimulated with 100 ng/mL LPS and submitted to the NIR light exposure protocol as described above. Following treatment, the cells were stained with 20 µM Rhod2 (ThermoFisher, Abingdon, UK) and incubated for 30 min at 37 °C, 5% CO_2_, and protected from light. The plates were read at 552/581 nm em/ex (FLUOStar Optima, BMG Labtech, Ortenberg, Germany). The mitochondrial Ca^2+^ levels were expressed as a percentage of the control without LPS.

### 2.9. Measurement of Mitochondrial Membrane Potential 

HEK-Blue™-hTLR4 cells were seeded on black 96-well clear-bottom plates at a seeding density of 1.25 × 10^4^ cells per well, stimulated with 100 ng/mL LPS and submitted to the NIR light exposure protocol as described above. Following treatment, cells were stained with 500 nM tetramethylrhodamine and ethyl ester (TMRE), (Sigma, Dorset, UK) and incubated for 30 min at 37 °C, 5% CO_2_, and protected from light. The plates were read at 549/575 nm em/ex (FLUOStar Optima, BMG Labtech, Ortenberg, Germany). The mitochondrial membrane potentials were expressed as a percentage of the control without LPS.

### 2.10. Measurement of Mitochondrial Function via SeaHorse MitoStress Assay

HEK-Blue™-hTLR4 cells were seeded on 24-well SeaHorse MitoStress Assay (Agilent, Wokingham, UK) cell plates at a seeding density of 1.25 × 10^4^ cells per well, stimulated with 100 ng/mL LPS and submitted to the NIR light exposure protocol as described above. Following treatment, the cells were washed and incubated in SeaHorse Assay medium supplemented with glucose (final concentration 4500 mg/L), sodium pyruvate and l-glutamine at pH 7.4 for 1 h at 37 °C. The oxygen consumption rate (OCR) was measured using the SeaHorse XF_E_ Flux Analyzer under basal conditions, followed by sequential injections of oligomycin (final concentration 2 µM), FCCP (3 µM), antimycin (1 µM) and rotenone (1 µM), allowing for the determination of the basal and maximal rates of mitochondrial respiration, as well as spare capacity, ATP production and non-mitochondrial respiration. Following the assay, OCR was normalized to total protein content, determined using the Bradford Assay and expressed as a percentage of the control without LPS.

### 2.11. Intracellular ROS Detection and Quantification

HAECs were seeded on cell observation chambers, stimulated with 1 μg/mL LPS and submitted to NIR light exposure for 10 min every 12 h over a total time of 48 h as described above. At indicated times during the NIR light exposure protocol, living HAECs were incubated in 40 mM potassium phosphate buffer (pH 7) containing 12.5 μM DCFH-DA (Molecular Probes by Thermo Fisher Scientific, Illkirch, France) for 15 min in a 95% air–5% CO_2_ incubator at 37 °C. Then, the cells were rinsed in the potassium phosphate buffer solution and observed immediately using an inverted Leica TCS SP5 microscope equipped with a 5% CO_2_–37 °C thermostatic observation chamber and using a 40× objective. Green fluorescence from DCFH-DA was excited at 488 nm wavelengths, and the emission fluorescence levels were detected using a photo-multiplier between 498 and 561 nm. Z series projections and the quantification of fluorescence intensity were performed using ImageJ software (National Institutes of Health, Bethesda, MD 20892, US; http://rsb.info.nih.gov/ij, accessed on 28 September 2023). The region of interest (ROI) corresponding to cells were drawn and mean fluorescent intensity (MFI) measured in each ROI.

### 2.12. ROS Analysis from Cell Extract via HPLC

HEK-Blue™-hTLR4 cells were seeded at a density of 10^6^ cells in 22.1 cm^2^ plates, stimulated with 100 ng/mL LPS for 24 h and submitted to NIR light exposure for 10 min. Immediately after NIR light exposure, the cells were washed once with 100 μM DTPA (diethylenetriaminepentaacetic acid) in PBS and incubated with 1 mL of PBS–100 μM DTPA–100 µM DHE (dihydroethidium) for 30 min at 37 °C. The cells were then harvested and washed twice with cold PBS-DTPA via centrifugation (1000 g, 5 min, 4 °C) and the cell pellets were resuspended in 0.5 mL cold acetonitrile, sonicated (10 s, 1 cycle at 8 W) and centrifuged (12,000× *g* for 10 min at 4 °C). The supernatants were harvested and dried under vacuum (Speed Vac Plus model SC-110A, Thermo Savant), and the dried oxidation products maintained at −80 °C in the dark until analysis. For analysis, the samples were resuspended in 200 µL PBS/DTPA and injected (100 µL per sample injection) into the HPLC system (Shimadzu, Marne la Vallée, France). The two main oxidation products derived from DHE were detected via fluorescence excitation at 396 nm/emission at 567 nm for 2-hydroxyethidium (2-E^+^OH) detection that reflected superoxide (O₂^•–^), and excitation at 510 nm/emission at 567 nm for ethidium (E^+^) detection that reflected hydrogen peroxide (H_2_O_2_). The analysis of the results was performed according to the protocol described in [39]. It is important to point out that all our experiments were performed in the absence of ambient light to avoid light interference with the infrared treatment. The results were normalized to total protein content, determined using the DC Protein Assay kit from Bio-Rad Laboratories (Mississauga, ON, Canada) and expressed as *n* moles of EOH or E/μ mole DHE. 

### 2.13. H_2_O_2_ versus NIR Light Treatment and SEAP Quantification for Monitoring TLR4/NF-κB Inflammatory Pathway in HEK-Blue™-hTLR4 Cells

HEK-Blue™-hTLR4 cells were seeded at a density of 2 × 10^40^ cells per well in 96-well plates and stimulated with 100 ng/mL of LPS to induce the TLR4/NF-κB inflammatory pathway. Negative control cultures were obtained by adding phosphate-buffered saline (PBS) at the same volume as the LPS. After LPS addition, the cell cultures were incubated for a further 16-h period before starting the relevant treatment (NIR light exposure or the addition of 50 μM H_2_O_2_). Both treatments were repeated once every 12 h, over a 48-h trial period. Each condition was repeated 5 times. The following day, inflammation was measured by determining the enzyme activity of the secreted alkaline phosphatase (SEAP) reporter gene, as described previously [33]. Briefly, 20 µL of cell-free supernatants from each of the five repetitions were mixed with 180 µL of QUANTI-Blue detection solution (Invivogen), which contains the AP colorimetric substrate, and incubated in accordance with the manufacturer’s specifications at 37 °C, 5% CO2, for 20 min in a fresh 96-well plate. The secreted alkaline phosphatase activity levels were measured at the absorbance of the detection solution at 620 nm using an Epoch microplate reader (BioTek, Winooski, VT, US) and were normalized to the total protein concentration (using the DC Protein Assay kit from Bio-Rad Laboratories, Mississauga, ON, Canada). Values from five duplicate wells were averaged to obtain a single experimental data point. The effects of the H_2_O_2_ and NIR light treatments are expressed as the percentage of inflammatory response achieved after LPS induction in untreated cells. 

### 2.14. Statistical Analysis

The data were analyzed using GraphPad Prism version 7.4.2 for Mac (GraphPad Software, La Jolla, CA, USA). The results are expressed as the mean ± standard error of the mean (SEM). The data were analyzed for normality using the Shapiro–Wilk test and the equality of group variances via the Brown–Forsythe test. The difference between treated and non-treated conditions was compared using two-way ANOVA analysis with the Tukey comparisons test. Differences were considered statistically significant with the following *p*-values: * *p* < 0.05, ** *p* < 0.01, *** *p* < 0.001.

## 3. Results

### 3.1. Exposure to NIR Light Reduces Inflammation in Primary Human Alveolar and Macrophage Cells

**Figure 1:**
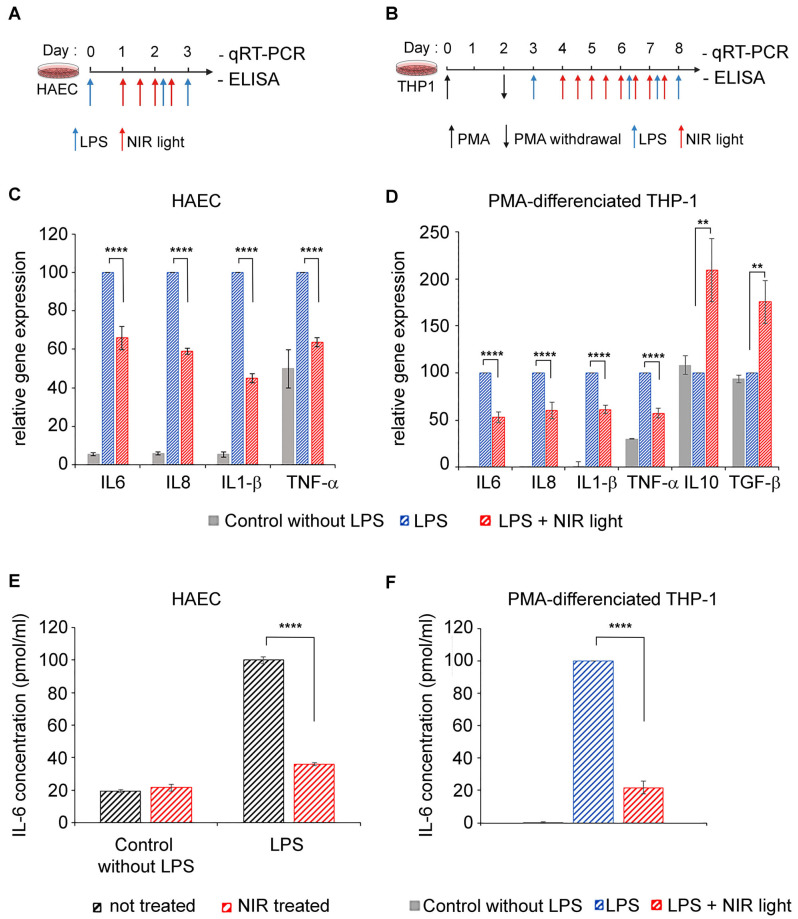
Effect of NIR light treatment on inflammatory cytokine and macrophage polarization. Schematic diagram for LPS-induced activation of TLR4/NF-κB inflammation pathway and NIR light treatment, in (**A**) HAECs and (**B**) PMA-differentiated THP-1 macrophage cell line. (**A**) HAECs were LPS-induced for the inflammatory response and maintained in an inflamed state following two additional LPS boosts (blue arrows), and subjected to brief 10-min intervals of NIR light exposure (red arrows), repeated once every 12 h, over a 48-h trial period. (**B**) PMA-differentiated THP-1 macrophage cell line was LPS-induced for the inflammatory response and maintained in an inflamed state following three additional LPS boosts (blue arrows) and subjected to brief 10-min intervals of NIR light exposure (red arrows), repeated once every 12 h, over a 96-h trial period. (**A**,**B**) Cells cultured without LPS induction were used as controls. For quantification of cytokine gene transcription via qRT-PCR, all cells were harvested 3 h after the last LPS boost. For ELISA quantification of IL6 secretion, cell supernatants were harvested 6 h after the last LPS boost. (**C**) Gene expression of inflammatory cytokines for HAEC control cells (grey bars) and LPS-induced HAECs with (red bars) or without (blue bars) NIR light treatment. (**D**) Gene expression of inflammatory and anti-inflammatory cytokines for THP-1 control cells (grey bars) and LPS-induced THP-1 with (red bars) or without (blue bars) NIR light treatment. Measurement of IL-6 protein release via ELISA, from (**E**) control and LPS-induced HAECs not treated (black bars) or NIR light-treated (red bars) and from (**F**) THP-1 control cells (grey bars) and LPS-induced THP-1 with (red bars) or without (blue bars) NIR light treatment. For (**C**,**D**), *n* = 4 to 6. For (**E**,**F**), *n* = 3. For (**C**–**F**), data are shown as standard error of the mean ± SEM; ** *p* < 0.01; **** *p* < 0.0001.

In severe cases of COVID-19, the cell types mediating dysregulated innate immunity, leading to a cytokine storm, are type II alveolar epithelial cells as well as resident and recruited macrophages [8,9]. We therefore tested the anti-inflammatory properties of near-infrared light on two physiologically relevant cell models: primary human alveolar type II cells (HAECs) and differentiated macrophage cell lines (THP-1). A TLR4/NF-κB pathway-dependent inflammatory response was induced by LPS in both cell cultures, at day 0 for HAECs (Figure 1A) and after differentiation at day 3 for THP-1 cells (Figure 1B). To maintain the inflammatory condition, LPS was added again on day 2 (to HAECs) and on days 6, 7 and 8 (to THP-1 cells). Beginning 24 h after LPS induction, inflamed cell cultures were exposed to NIR light at a fluence of 6 W/m^2^ for 10 min. This exposure treatment was repeated at 12-h intervals over two days (for a total of four exposures) for HAECs and over four days (for a total of eight exposures) for THP-1 cells. Twelve hours after the last NIR light exposure, the cells were challenged with an ultimate LPS boost and the inflammatory response measured via qRT-PCR cytokine gene expression analysis (Figure 1C,D) as well as via an ELISA of IL-6 secretion (Figure 1D). 

In the HAEC and THP-1 cultures, LPS induced an increase in the inflammatory cytokine gene expression of IL-6, IL-8, IL-1β and TNF-α (Figure 1C,D; compare gene expression of control and LPS-stimulated cells). In macrophages, these expression profiles are characteristic of pro-inflammatory (M1) polarization. However, NIR therapeutic light treatment resulted in a significant reduction in all of the proinflammatory cytokine gene markers tested (Figure 1C,D; compare LPS-stimulated with LPS-stimulated + NIR cell samples). Most interestingly, the expression of anti-inflammatory cytokines IL10 and TGF-β was actually increased following exposure to NIR light (Figure 1D). This indicates that NIR light exposure not only counteracts increased inflammatory cytokine transcription induced via the TLR4-dependent innate immune system, but it also promotes the phenotypic switch of macrophages from pro-inflammatory M1 toward the anti-inflammatory M2 phenotype involved in inflammation resolution and repair [40,41].

The IL-6 cytokine has been identified as the most abundant inflammatory cytokine accumulating in COVID-19 patients and the most harmful in the progression of the disease [42,43]. We therefore examined the levels of secreted IL-6 in culture supernatants of both HAECs and THP-1 cells via ELISA (Figure 1E,F). Control HAECs that had not been LPS-stimulated produced only a minor level of secreted IL-6. Subsequent NIR light exposure did not change the levels of IL-6 secretion detected in these control cells (see Figure 1E). This indicates that NIR light treatment does not affect the response pathway of non-inflamed cells. LPS stimulation induced a significant increase in IL-6 secretion (Figure 1E), as expected. In this case, however, NIR light exposure had a dramatic effect on IL-6 secretion. In fact, the levels of IL-6 secretion were reduced close to the background control levels, indicating near-total inhibition of the hyperinflammatory response in these cells (Figure 1E). Similar results were obtained in the case of macrophage THP-1 cell cultures, where NIR light treatment resulted in a five-fold reduction in the levels of secreted IL-6 (Figure 1F). Significantly, the inhibition of IL-6 secretion (70–80%) was more pronounced than the inhibition of IL-6 gene expression (30–50%—Figure 1C,D), suggesting that NIR light counteracts the cellular inflammatory pathway at multiple signaling steps and acts in just a handful of days.

To exclude the possibility that NIR light effects might be due to an elevation in temperature, we measured real-time cell temperature fluctuation once per second during the 10 min NIR light exposure, as well as 20 min before the NIR light was switched on and 20 min after it was switched off. The results described in Supplementary Figure S1 show a stable temperature of 36.81 °C ± 0.07, remaining stable up to 20 min after the end of NIR light exposure. This clearly indicates that NIR exposure does not increase the temperature of the cellular medium. Therefore, the effects of NIR exposure are exclusively caused by light.

### 3.2. Exposure to NIR Light Reduces Inflammation Triggered by the SARS-COV-2 Spike Protein

For reasons of convenience, we and others have used LPS, an archetypal PAMP agonist, for in vitro or in vivo studies requiring stimulation of the TLR4 signaling pathway. This seems reasonable as in past studies, the SARS-CoV-2 spike protein has been shown to bind directly to TLR4, triggering inflammation identical to that in cells stimulated by other PAMP- or DAMP-type TLR-4 ligands [14,15,16,44]. To verify that our approach was indeed valid for the treatment of the SARS-CoV-2-induced inflammation, we examined whether the anti-inflammatory effects of NIR light exposure on our cell cultures also occurred when inflammation was triggered by the viral spike protein. 

**Figure 2:**
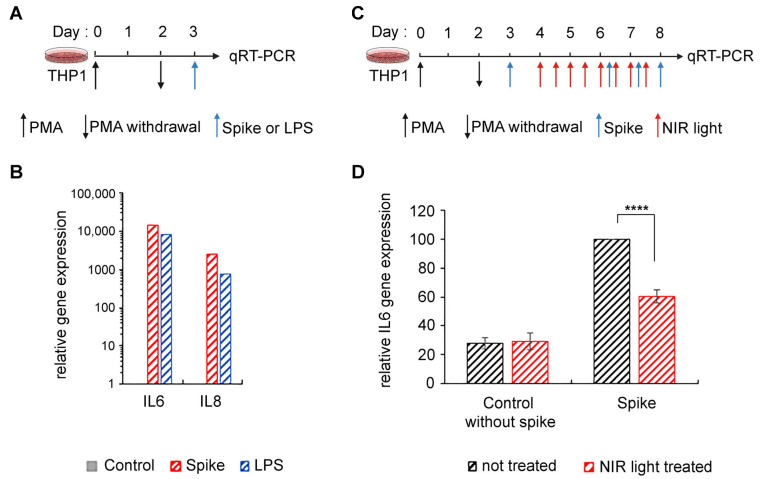
Effect of NIR light treatment on SARS-CoV-2 spike-induced inflammation in macrophage THP-1 cell line. (**A**) Schematic diagram of the experiment: PMA-differentiated THP-1 macrophage cell line was activated by SARS-CoV-2 spike protein or LPS for 3 h and harvested for qRT-PCR to determine (**B**) spike (red bars) and LPS (blue bars) induction of IL6 and IL8 gene transcription compared to control cells without activation (grey bars). (**C**) Schematic diagram for spike-induced activation and NIR light treatment in PMA-differentiated THP-1 macrophage cell line. Cells were spike-induced for the inflammatory response and maintained in an inflamed state by three additional spike-boosts (blue arrows) and subjected to brief 10 min intervals of NIR exposure (red arrows), repeated once every 12 h, over a 96 h trial period. Cells were harvested 3 h after the last spike boost and subjected to (**D**) IL6 gene expression via qRT-PCR. For (**D**), *n* = 3, and data are shown as standard error of the mean ± SEM; **** *p* < 0.0001.

We synthesized the SARS-CoV-2 S protein in a eukaryotic expression system in order to achieve appropriate post-translational modification with *N*-linked glycans, which protrude from the trimer surface [45,46]. This method also excluded the possibility of LPS contamination. Using the SARS-CoV-2 S ectodomain to trigger inflammation (Figure 2A), we saw marked stimulation of proinflammatory cytokine expression in differentiated THP-1 macrophages (Figure 2B), consistent with prior reports [14,15]. Moreover, this stimulation was equivalent to the levels obtained after LPS exposure (Figure 2B). We next examined the effect of NIR light on the inflammatory response in these macrophages (Figure 2C). The effect of NIR light treatment on the expression of inflammatory cytokine gene IL-6 was virtually identical to the results obtained using LPS as an inducer (Figure 2D). 

In sum, NIR light treatment is effective in downregulating the TLR4-dependent inflammatory response irrespective of how it was induced, whether by the SARS-CoV-2 S protein or by other inducers that activate the TLR4/NF-κB pathway (such as LPS). Therefore, in this study, LPS is used interchangeably with the SARS-CoV-2 S as an elicitor to trigger the inflammatory response.

### 3.3. Exposure to NIR Light Stimulates Mitochondrial Metabolic Activity

**Figure 3:**
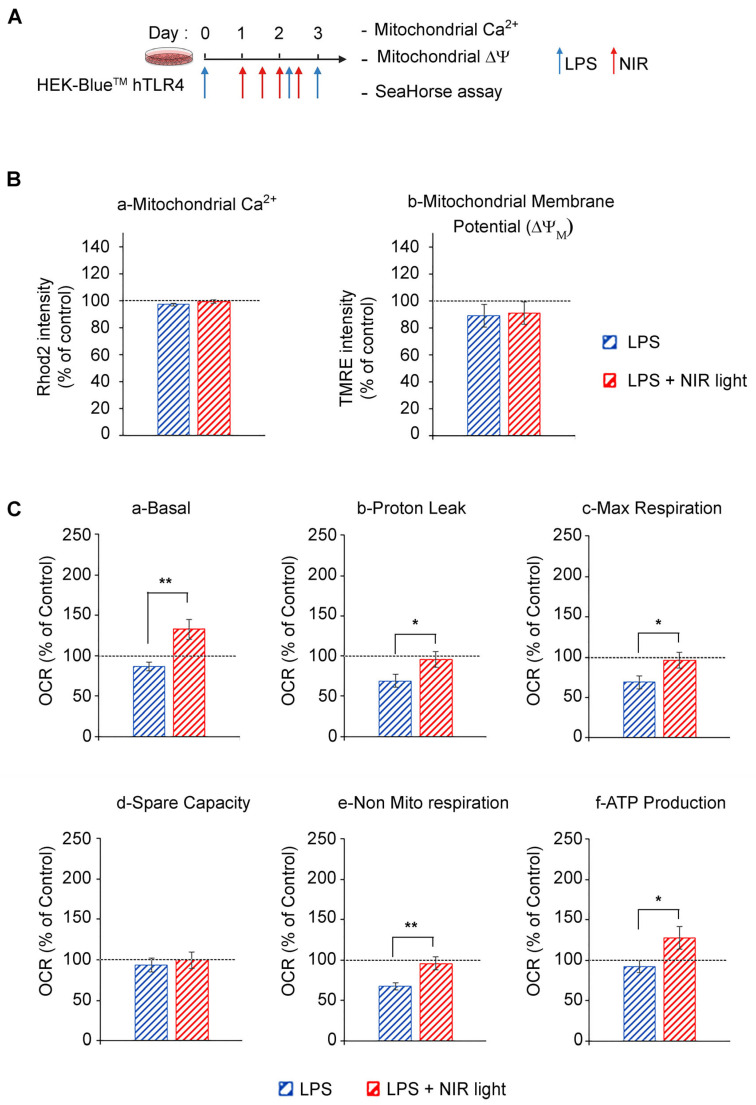
Effect of NIR light on mitochondrial functions. (**A**) Schematic diagram of the experiment: HEK-Blue^TM^ hTLR4 cell cultures were LPS-induced for the inflammatory response (blue arrows) and subjected to brief 10-min intervals of NIR light exposure (red arrows), repeated once every 12 h, over a 48-h trial period. After the last LPS boost, cells were subjected to (**B**) measurement of mitochondrial calcium and membrane potential as well as (**C**) measurement of oxygen consumption rate using SeaHorse MitoStress Assay, under basal condition (**a**) and after addition of inhibitors (Oligomycine, FCCP, antimycine and rotenone) to deduce the values of proton leak (**b**), maximal respiration (**c**), spare capacity (**d**), non mitochrondrial respiration (**e**) and ATP production (**f**). For (**B**,**C**), *n* = 7, and data are shown as standard error of the mean ± SEM; * *p* < 0.1; ** *p* < 0.01.

It is widely accepted that mitochondrial cytochrome C oxidase from complex IV of the electron transport chain (ETC) is a cellular photoacceptor of photobiomodulation light (red and NIR wavelengths), resulting in the stimulation of mitochondrial oxidative phosphorylation (OXPHOS), the enhanced production of ATP and a transient increase in ROS in a number of experimental systems [23,47,48,49]. We accordingly verified whether NIR light exposure had an effect on mitochondrial activity in cell cultures induced for the TLR4-dependent inflammatory response (Figure 3). In this analysis, we used HEK-Blue^TM^ hTLR4 model cell cultures. After the induction of the inflammatory response using LPS, the cells were subjected to brief 10-min intervals of NIR light exposure, repeated once every 12 h, over a 48 h trial period (four NIR light exposure treatments in all) as described in Figure 3A and previously [32,33].

We next measured the effect of NIR light on mitochondrial function using the SeaHorse assay, in which oxygen consumption rates (OCR) were measured before and after the addition of selective inhibitors of respiratory chain complexes I to V. LPS-induced inflammation triggered a decrease in basal and maximal mitochondrial respiration, proton leakage and ATP production as compared to the control cells, indicating OXPHOS dysfunction consistent with TLR4/TRAF6 signaling activation that induces ETC complex I disassembly [50] (Figure 3C). By contrast, NIR light exposure increased basal mitochondrial respiration, proton leak and maximal respiration, compared to non-exposed cells (Figure 3C(a)–(c)), indicating an upregulation of OXPHOS activity, confirmed by the increased production of ATP (Figure 3C(f)). Importantly, there was no change in mitochondrial reserve capacity, indicating that the NIR light effect was likely to stimulate activation of the mitochondrial respiratory chain enzymes themselves. In sum, NIR light exposure significantly improves mitochondrial respiration and ATP production in cells induced for the TLR-4-dependent inflammatory response after a treatment interval of several days. This can lead to both direct and indirect anti-inflammatory effects (see below).

### 3.4. A Single Bout of NIR Light EXPOSURE Triggers Increased Cellular ROS in Inflamed Cell Cultures but Not in Control Cells

Given that mitochondria were responsive to extended periods of NIR light exposure, we next looked for more immediate consequences of NIR light exposure on the mitochondria which might explain the anti-inflammatory effects. Given that NIR light exposure stimulates the mitochondrial electron transport chain, we investigated whether this might trigger an immediate, transient increase in reactive oxygen species. ROS are highly reactive oxygen radical derivatives that have long been considered deleterious to cells; however, stimulation with mild, controlled bursts of ROS can actually be beneficial to cell growth and survival. In fact, ROS at moderate concentrations act as physiological signaling intermediates to induce the very cellular protective mechanisms that counteract the damaging effects of excess ROS (oxidative stress). In particular, the mild stimulation of ROS has been shown to be beneficial for controlling inflammation (reviews in [35,36,51]). This type of response mechanism, where low doses of a potential toxin induce resistance to a higher damaging dose, is known as a ‘hormetic’ mechanism, and is typical for many toxins and certain hormonal signaling pathways [52].

**Figure 4:**
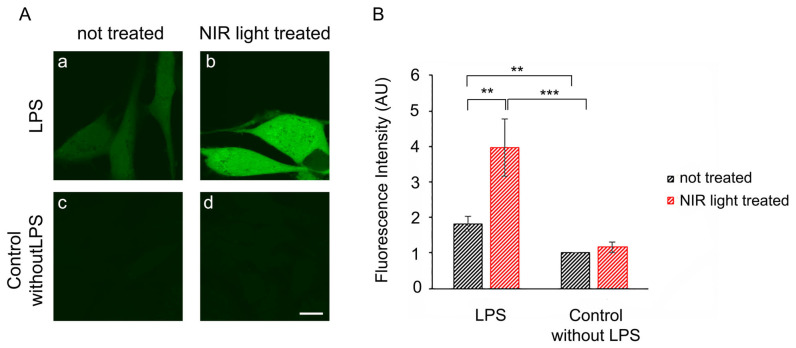
Immediate effect of NIR light on ROS production. (**A**)Twenty-four hours after seeding and being LPS-induced (**a**,**b**) or not LPS-induced (**c**,**d**) for activation of inflammation, living HAECs were dye-labeled with DCFH-DA during the 10 min of NIR light exposure (**b**,**d**) or during 10 min without NIR light exposure (**a**,**c**) and observed immediately afterwards using an inverted Leica TCS SP5 confocal microscope. Images (**a**–**d**) show a single confocal z section that crosses the nucleus and are representative of 3 independent experiments (*n* = 3). Scale bar is 10 μm. (**B**) Fluorescence intensities were measured cell-by-cell on z-projected images using ImageJ. Average values were obtained from 3 independent experiments (*n* = 3) and are shown as standard error of the mean ± SEM; ** *p* < 0.01; *** *p* < 0.001.

We therefore investigated whether the periodic synthesis of ROS may be the underlying cause of the anti-inflammatory effects of NIR light. We first determined whether a single 10-min bout of NIR light exposure could trigger the formation of ROS in cultured cells. HAEC cells that had been induced with LPS for 24 h were exposed to 10 min of NIR light in the presence of the dye DCFH-DA, which fluoresces in the presence of ROS. Immediately after the light pulse, ROS fluorescence was assayed via inverted confocal living cell microscopy using established methods [53]. The results showed that in uninflamed control cells (Figure 4A, panel (c)), there were only basal, low concentrations of cellular ROS. These cells, furthermore, showed no significant change in ROS after a single bout of exposure to NIR light (Figure 4A, compare panels (c) and (d)). By contrast, cells with LPS-induced inflammation showed elevated levels of ROS (Figure 4A, panel (a)) as compared to the control cells (panel (c)). After exposure to NIR, these cells showed a dramatic and immediate increase in fluorescence (Figure 4A, compare panels (a) and (b)), more than doubling the total cellular concentration of ROS, as confirmed through quantification (Figure 4B). 

In sum, a single bout of NIR light exposure stimulated a significant, transient increase in ROS in inflammation-induced cells, but had negligible effects on control cell cultures.

### 3.5. Multiple Successive Bouts of NIR Light Exposure Cause a Decrease in Intracellular ROS

**Figure 5:**
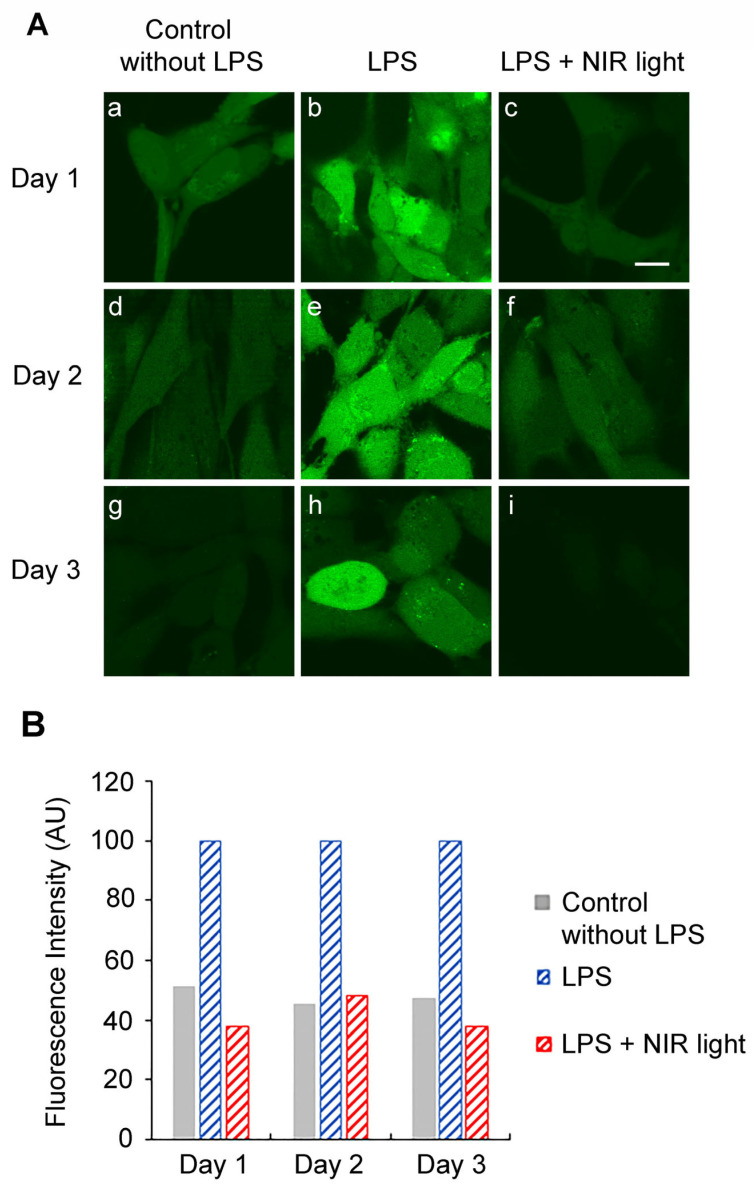
Effect of long-term and repetitive NIR light exposure on ROS production. (**A**) At day 0, HAECs were seeded and LPS-induced (**h**,**i**) or not induced (**a**–**g**) for activation of inflammation and subjected (**c**,**f**,**i**) or not subjected (**a**,**b**,**d**,**e**,**g**,**h**) to brief 10 min intervals of NIR light exposure, repeated once every 12 h, over a 48 h trial period according to the experimental procedure depicted in Figure 1A. At day 1 (**a**–**c**), day 2 (**d**–**f**) or day 3 (**g**–**i**) post seeding, that correspond respectively to 6 h, 6 h and 18 h after the last NIR light exposure, living HAECs were dye-labeled with DCFH-DA for 10 min and observed using an inverted Leica TCS SP5 confocal microscope. Control cell cultures not exposed to NIR light were treated in an identical manner. Scale bar is 10 μm. (**B**) Fluorescence intensities were measured cell-by-cell on z-projected images using ImageJ.

To downregulate the TLR4-dependent inflammatory response, multiple NIR light exposures are necessary over a 2-day period. We therefore measured the levels of cellular ROS on subsequent days in the course of an entire treatment protocol (Figure 1). For this experiment, HAECs were induced with LPS at day 0, maintained in an inflamed state and subjected to brief 10-min intervals of NIR light exposure, repeated once every 12 h, over a 48-h trial period (Figure 1A). As a control condition, we measured ROS in cells that had not been induced for inflammation (No LPS) and inflamed cells that had not been exposed to NIR light treatment (LPS) at the same time (Figure 5). On days 1 and 2 of this treatment, HAECs were assayed for levels of cellular ROS 6 h after NIR light exposure, using DCFH-DA dye fluorescence imaging via confocal microscopy in living cells (Figure 5). On day 3, the ROS levels were assayed 18 h after NIR light exposure and 6 after the last LPS boost. 

On day 1, there were elevated levels of cellular ROS in HAECs with LPS-induced inflammation (Figure 5A, panel (b)) as compared to the uninflamed control cells (Figure 5A, panel (a)), consistent with our previous results (Figure 4). However, the LPS-induced cells showed a significant decline in cellular ROS measured 6 h after just a single NIR light treatment, reaching levels comparable to those in the non-inflamed control cells (Figure 5A; compare panels (b) and (c)). This was in marked contrast to the dramatic increase in ROS seen immediately after NIR light exposure (Figure 4, compare panels (a) and (b)). In other words, the transient increase in ROS induced by NIR light exposure was followed by a drop to levels even below those prior to NIR light exposure.

The relevant species of ROS that were downregulated by NIR light exposure were further characterized via HPLC analysis of cellular lysates, performed according to the protocols in [39]. These downregulated species were shown to consist primarily of H_2_O_2_, with a minor component of superoxide radical O_2_^• – ^(Supplementary Figure S2). Both of these levels were significantly higher in inflamed cell cultures, and decreased dramatically within 1 h after NIR light exposure.

Similar results were seen using imaging techniques for days 2 and 3, in which ROS levels were assayed, respectively, 6 and 18 h after the NIR light exposure period. In every instance, the levels of ROS in the NIR light-treated cells (panels (f) and (i)) were decreased to the levels in the control non-inflamed cells (panels (f) and (i)). In addition, at day 3, our results show that the accumulation of NIR light exposure can efficiently prevent ROS induction after a last, ultimate LPS boost. 

In sum, NIR light causes a transient increase in cellular ROS (Figure 4), but the net effect is to reduce the levels of cellular ROS in inflamed cells almost down to background levels (Figure 5).

### 3.6. NIR Light Exposure Modulates Expression of Genes for ROS-Scavenging Enzymes

**Figure 6:**
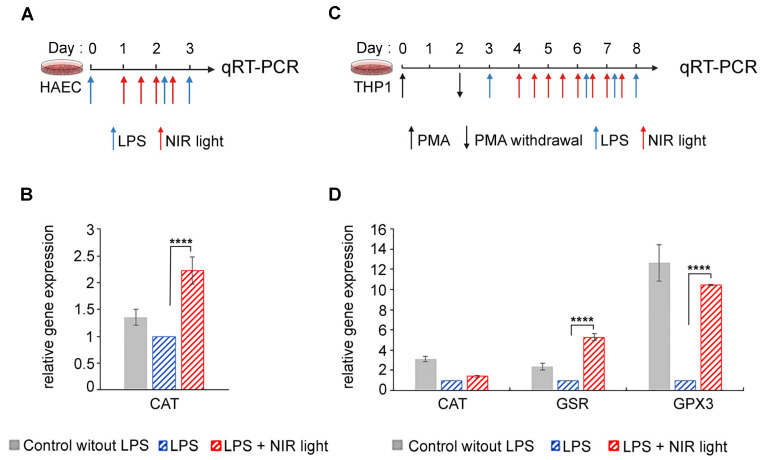
Effect of NIR light treatment on increased expression of antioxidant genes. Schematic diagram for LPS-induced activation of TLR4/NF-κB inflammation pathway and NIR light treatment, in (**A**) HAECs and (**C**) PMA-differentiated THP-1 macrophages cell line. (**A**) HAECs were LPS-induced for the inflammatory response and maintained in an inflamed state by two additional LPS boosts (blue arrows) and subjected to brief 10 min intervals of NIR light exposure (red arrows), repeated once every 12 h, over a 48 h trial period. (**B**) PMA differentiated THP-1 macrophages cell line were LPS-induced for the inflammatory response and maintained in an inflamed state by three additional LPS boosts (blue arrows) and subjected to brief 10 min intervals of NIR light exposure (red arrows), repeated once every 12 h, over a 96 h trial period. (**A**,**B**) Cells cultured without LPS induction were used as controls. For quantification of antioxidant gene expression via qRT-PCR, all cells were harvested 3 h after the last LPS boost. (**B**) Gene expression of catalase (CAT) for HAEC control cells (grey bars) and LPS-induced HAECs with (red bars) or without (blue bars) NIR light treatment. (**D**) Gene expression of catalase (CAT), glutathione reductase (GSR) and glutathione peroxidase 3 (GPX3) for THP-1 control cells (grey bars) and LPS-induced THP-1 with (red bars) or without (blue bars) NIR light treatment. For (**B**), *n* = 8 and (**D**), *n* = 3 to 6; **** *p* < 0.0001.

One possible explanation for this apparent contradiction is that the increase in ROS induced immediately subsequent to NIR light exposure may, in turn, strongly induce antioxidant mechanisms, resulting in the dramatic subsequent lowering of cellular ROS levels, which persists over the longer term. To test this possibility, we measured the expression of genes for several antioxidant enzymes (GPX1, SOD2, CAT, GSR and GPX3) in both HAECs and THP-1 cells submitted to the NIR light protocols described in Figure 6A,B, respectively.

In the HAEC cell cultures, we observed a marked increase in catalase (CAT) transcription levels after NIR light treatment (Figure 6B). This enzyme scavenges H_2_O_2_ and thereby reduces the cellular concentrations of ROS. The induction of ROS-scavenging enzymes would fully explain the dramatically reduced levels of cellular ROS subsequent to NIR light exposure in HAECs.

In the case of differentiated macrophage THP-1 cell cultures, changes were observed in the expression of the antioxidant enzymes CAT, GSR and GPX3 subsequent to NIR light exposure (Figure 6D). Oddly, all of these enzymes were actually downregulated in inflamed cells (LPS) as compared to the non-inflamed control cultures (No LPS), suggesting that elevated levels of cellular ROS in this cell type do not stimulate the synthesis of antioxidant genes, but rather, the opposite. By contrast, NIR light exposure caused an increase in the expression of both GSR- and GPX3 ROS-scavenging enzymes (Figure 6D), in keeping with the dramatic reduction in ROS as a result of NIR light exposure.

In sum, rapid upregulation of ROS-scavenging enzymes occurs in response to the transient increase in ROS triggered by NIR light exposure, leading to an overall net decrease in cellular ROS.

### 3.7. Transient Increase in Cellular ROS Causes Anti-Inflammatory Effects of NIR Light Exposure

The ultimate question is whether there is indeed a causal relationship between changes in cellular ROS, triggered by NIR light through its effect on mitochondrial cytochrome C oxidase, and a decrease in cytokine production. Such a mechanism would identify ROS synthesis as a physiologically relevant mechanism for the anti-inflammatory effects of NIR light.

**Figure 7:**
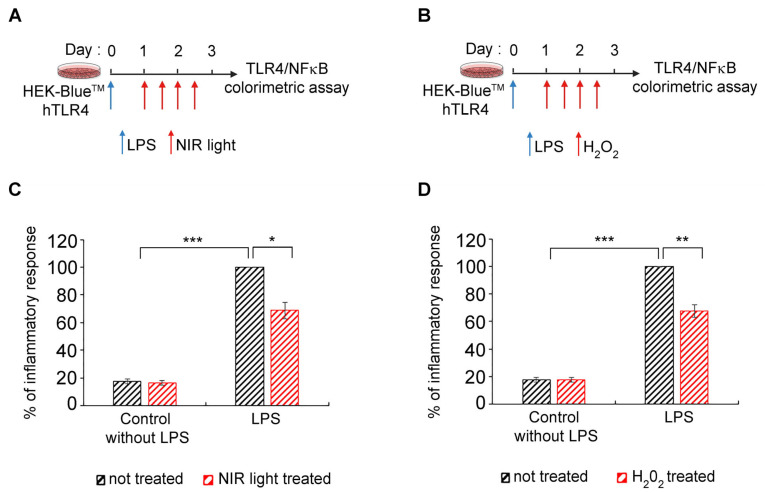
Effect of added H_2_0_2_ on TLR4/NF-κB inflammation pathway. Schematic diagram for LPS-induced activation of TLR4/NF-κB inflammation pathway and effect of NIR light (**A**) or 100 μM H_2_O_2_ (**B**) treatment in HEK-Blue^TM^ hTLR4 cells. HEK-Blue^TM^ hTLR4 cells were LPS-induced for the inflammatory response and maintained in an inflamed state via two additional LPS boosts (blue arrows) and subjected to (**A**) brief 10-min intervals of NIR light exposure or (**B**) 100 μM H_2_O_2_ treatment. Both treatments (red arrows) were repeated once every 12 h, over a 48-h trial period. ((**C**,**D**)) Measurement of inflammation via colorimetric assay in control without LPS and LPS-induced HEK-Blue^TM^ hTLR4 not treated (black bars) or treated (red bars) with NIR light (**C**) or H_2_O_2_ (**D**). *n* = 5, * *p* < 0.1; ** *p* < 0.01; *** *p* < 0.001.

To address this question, we used HEK-Blue™-hTLR4 model cell cultures, in which inflammation can be detected using a simple colorimetric assay [54,55], and which show an anti-inflammatory response to NIR light exposure, similarly to primary cell cultures [32,33]. As a control condition, LPS-induced cells were submitted to the NIR light treatment protocol as described in Figure 7A and in [32,33]—see also Figure 1A. As test conditions, parallel cultures were used under the same growth regimen except that, instead of NIR light exposure, H_2_O_2_ in solution was physically added to the culture medium. The timing of the H_2_O_2_ additions was identical to the timing of NIR light exposure in the control cultures (Figure 7B). The progression of the inflammatory response was compared in the two conditions (Figure 7C,D).

These results provide conclusive evidence of a causal relationship, at the cellular level, between the anti-inflammatory mechanism of NIR light exposure and an induced transient increase in ROS. 

## 4. Discussion

In this work, we provide support for the use of photobiomodulation therapy against lethal dysregulated inflammation in present and future COVID epidemics. We also provide the primary mechanism of PBM’s effects through the transient induction of ROS. 

### 4.1. Effect of PBM on the Modulation of ROS and Mitochondrial Activity

Our results show consistently elevated levels of ROS in inflamed cell cultures as compared to the control cells (Figure 4 and Figure 5). We have, moreover, identified the major species, via HPLC analysis, as comprising both (O_2_^• – ^) superoxide and hydrogen peroxide (H_2_O_2_), both of which have known physiological roles. These levels decreased within an hour of NIR light exposure, reaching near-background levels after only a few days (Figure 4 and Figure 5). There was concurrent improvement in mitochondrial function, respiration and ATP synthesis as a result of NIR light treatment (Figure 3), consistent with past reports [23,56]. Because the breakdown of mitochondrial function and the exacerbation of inflammation can also occur through the leakage of mitochondrial factors acting as DAMPS [57], improved mitochondrial function may also promote anti-inflammatory effects by indirectly reducing the leakage of pro-inflammatory factors.

By contrast, the effect of NIR light on cells immediately after exposure was to induce an actual increase in cellular ROS. This change was considerable, literally doubling the concentration of H_2_O_2_ in the cells within a few minutes (Figure 4 and Figure 5). NIR light is known to be absorbed by the chromophores of the mitochondrial enzyme CCO (cytochrome C oxidase), which, in turn, promotes enzymatic electron transfer in the mitochondrial inner membrane [58]. An accompanying release of mitochondrial reactive oxygen species thereby provides a physical explanation for our results of a transient oxidative burst [59]. 

### 4.2. Causal Relationship between Anti-Inflammatory Effects of PBM and ROS

ROS is known to play a dual role in cellular growth and metabolism. On the one hand, elevated concentrations of ROS lead to molecular damage and oxidative stress; on the other hand, at lower concentrations, they play a central role in redox signaling and stress resistance through the promotion of cellular defense and repair mechanisms [60]. As a result, it has been speculated that the transient elevation in ROS induced by PBM might mediate its therapeutic effects through the induction of cellular anti-inflammatory mechanisms [23]. The fact that the innate immune signaling pathways are responsive to ROS [61,62,63,64] has lent support to this idea. 

In this work, we show for the first time that there is a direct causal relationship between the transient formation of ROS induced by NIR and the anti-inflammatory effects of NIR light on the innate immune system. When hydrogen peroxide (H_2_O_2_, the most physiologically relevant cellular ROS species) was introduced into human cell cultures at exactly the same time intervals as the PBM treatments, the exogenous H_2_O_2_ could fully replace NIR light in the downregulation of the inflammatory response. Moreover, the anti-inflammatory effects of added H_2_O_2_ on cytokine synthesis and secretion were of the same order as those of PBM (Figure 7A). Therefore, the transient increase in cellular ROS induced by PBM is both necessary and sufficient to counteract the excessive hyperinflammatory response in the case of the TLR-4 dependent cell signaling pathway. This might provide the missing physiological mechanism for how PBM can treat inflammation, with significant therapeutic implications (see below).

### 4.3. Underlying Mechanism of Photobiomodulation: Hormesis

There have been numerous reports of bell-shaped or so-called ‘hormetic’ dose–response curves for PBM therapy (see, e.g., [23,65]). This means that, at very low light doses, there is no therapeutic effect on the biological system; at intermediate light doses, there is an optimum effect; and at even higher light doses, there is again no therapeutic effect. In some cases, when the signal intensity is increased to an even greater extent, the biological effect may even be the opposite to that at low doses. These so-called ‘hormetic’ effects are common in toxicology (see, e.g., [66]). They are also seen for ROS, where low doses are beneficial, intermediate doses have no effect, and very high doses are detrimental to cells (see, e.g., [60,67,68]). 

This notion of hormesis, together with the mechanistic requirement for an oxidative burst, could explain the PBM exposure parameters for downregulation of the inflammatory response pathway. Specifically, we empirically found that exactly 10 min of 730 nm light, at an intensity 1–2 W/m^2^ or greater, gave an optimal anti-inflammatory effect [32]. Even 5 min less or 5 min more of illumination were relatively ineffective. Increasing the light intensity above the threshold (1–2 W/m^2^) had no added therapeutic benefit, but did no harm either. 

**Figure 8:**
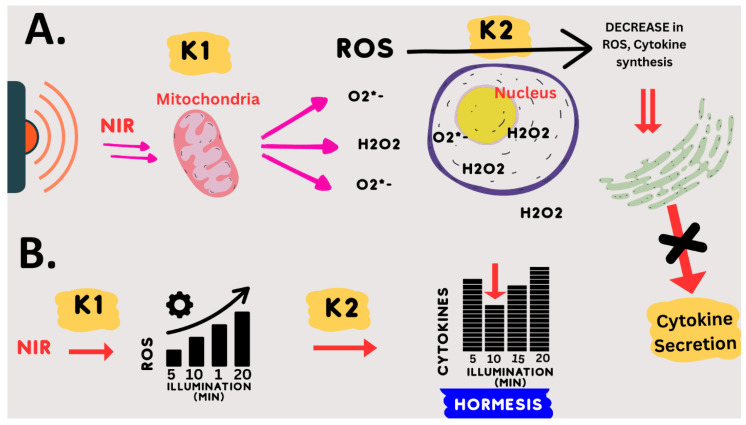
Model of Effect of NIR light exposure on human cells. (**A**) NIR light is absorbed by the mitochondria and induces the enzymatic formation of ROS at a rate (K1) dependent on the light intensity until a saturating intensity is reached (2 W/m^2^). These ROS species are primarily H_2_O_2_ and O_2_^• – ^. These, in turn, stimulate ROS-scavenging enzymes and downregulate the expression of inflammatory genes via multiple cellular mechanisms controlled by modulating the concentration of ROS (K2). The ensuing abrupt decline in overall cellular ROS, as well as an abrupt decline in cytokine biosynthesis and secretion, short-circuits the cytokine storm effect. (**B**) The hormetic mechanism occurs at the level of ROS signaling. The NIR light stimulation of ROS occurs via an enzymatic mechanism (K1) that reaches saturation at 2 W/m^2^ light intensity (32). At this light intensity and higher, the amount of ROS formed in response to NIR light is directly proportional to the illumination time and not to the light intensity (Michaelis–Menten kinetics). A transient increase in ROS downregulates cytokine synthesis via a hormetic mechanism, whereby there is no effect at 5 min, a maximal effect at 10 min, and little to no effect at 15 or 20 min of exposure time. This inverse curve is typical of hormetic mechanisms for ROS signaling and is determined by sensitivity (k2) to the concentration of ROS.

This dosing protocol can be mechanistically explained by the following model: (1) NIR light exposure (at 730 nm) directly stimulates mitochondrial enzymes CCO and others to form ROS. (2) These enzymatic reactions (k1) reach their maximum rate at an intensity of 2 W/m^2^ NIR illumination (saturation). This explains why higher light intensities will not increase the synthesis of ROS. (3) The optimum concentration of ROS is formed after exactly 10 min of saturating enzyme activity (k1); (4) less light exposure time (e.g., 5 min) results in insufficient ROS, whereas (5) increased light exposure time (e.g., 15 min) results in too much ROS. Due to the hormetic effect of ROS on downregulating inflammation, both too much and too little ROS are ineffective in stimulating the anti-inflammatory response. This model is summarized in Figure 8.

### 4.4. Considerations of Wavelength and Exposure Parameters

The wavelength of 730 nm used in this study was selected empirically on the basis of optimal anti-inflammatory effects on cell cultures. However, it is neither widely used nor widely commercially available. This may be because, historically, the PBM action spectra determined for phenomena like RNA synthesis or cell adhesion did not show peaks of effectiveness in the 700–750 nm spectral range [59]. This no doubt discouraged the further use of this wavelength range. However, 730 nm has not only proven highly effective for treating inflammation in culture [32] but has two additional advantages over other wavelengths. Firstly, unlike the popular red light (e.g., 630 or 660 nm) wavelength, which does not penetrate the skin, the 730 nm wavelength penetrates into the body to a depth of more than 6 cm at the therapeutic minimum intensity required for the treatment of lung inflammation [32,33]. It can, moreover, do so without unduly heating the skin or causing discomfort. Therefore, if administered to both sides of the chest simultaneously, this wavelength should penetrate throughout the lung. Secondly, 730 nm is therapeutically effective at light intensities up to two orders of magnitude above the minimum threshold intensity needed to achieve anti-inflammatory effects [32,33]. This is critical as lungs are voluminous organs in which a significant light gradient will be established by external illumination. Therefore, it is necessary that both high-intensity light (reaching lung tissue near the skin surface) and low-intensity light (reaching lung tissue deep inside the body) must be equally therapeutically effective. 

This seems not to be the case with other wavelengths in common usage. For example, 810 nm light, which also has anti-inflammatory effects in cell culture, shows a biphasic dose–response curve. This means that at light intensities either below or above a certain optimum intensity, the light is ineffective in treating the inflammation [23]. As a consequence, such a wavelength applied externally could only effectively treat a narrow slice of lung tissue in a patient.

### 4.5. PBM Therapy for the Next COVID Epidemic

The advantage of PBM as a therapy for COVID epidemics is that it can be implemented immediately, non-invasively, with no negative side effects and without compromising the whole immune system (it targets only the lungs). Therefore, it can be combined with anti-viral agents, boosting the immune system and targeting the pathogen. PBM would moreover be equally effective for any SARS-CoV-2 variant.

Photobiomodulation is currently approved by the US Food and Drug Administration for the treatment of non-lethal conditions such as chronic pain, wound healing, alopecia, wrinkles, bone healing, psoriasis, lupus, post-operative edema and osteoarthritis. However, it has never been approved for the treatment of life-threatening conditions caused by infectious disease.

Our work here has shown that under an optimized exposure condition, and in the relevant cell types (primary human lung and macrophage cells), the effect of NIR light on downregulating the SARS-CoV-2 spike-induced inflammatory response is both dramatic and rapid. Our work has, in addition, addressed one of the remaining obstacles to the acceptance of PBM therapy, by demonstrating an underlying mechanism that could be relevant to explaining the anti-inflammatory effects of PBM. 

In support of our results, the recent COVID-19 pandemic has prompted several small-scale studies and case reports using forms of photobiomodulation therapy to treat acute lung inflammation in hospitalized COVID-19 patients [28,29,30,31,69]. Although their PBM exposure conditions varied greatly, they showed benefits to the patients, as demonstrated by improved subjective health, decreases in blood cytokine levels, and improved respiratory capacity.

We will require large-scale, double-blind, placebo-controlled clinical trials to validate these promising findings and develop a treatment recognized by the public health authorities.

### 4.6. Treating Other Pathologies

Our present work shows the efficiency of NIR light exposure in downregulating the TLR4/NF-κB inflammatory pathway, which is shared by other viral pathologies in addition to COVID-19. These include infections with respiratory syncytial virus (RSV), influenza A virus (IAV), Ebola virus (EBOV) and Dengue virus (DENV) [70], all associated with serious morbidity and mortality. Moreover, during sepsis caused by Gram-negative bacteria, excessive TLR4/NF-κB activation may result in fatal septic shock [13]. 

In the face of increasingly frequent viral and bacterial outbreaks, there is an urgent need to develop new therapeutic concepts that are more effective and broader-spectrum than conventional treatments. NIR light exposure, together with an understanding of its underlying mechanism of action, could provide a virtually ideal solution to this problem.

## Data Availability

All data are available in the main text or the Supplementary Materials.

## Supplementary Materials

The following supporting information can be downloaded at: https://www.mdpi.com/article/10.3390/antiox12101824/s1, Figure S1: Measure of real-time cell temperature fluctuation during NIR exposure; Figure S2: HPLC analysis of ROS products after NIR light exposure; Table S1: List of primers used for quantitative real-time PCR.
