# Supplementary material for: Near-Infrared Light Exposure Triggers ROS to Downregulate Inflammatory Cytokines Induced by SARS-CoV-2 Spike Protein in Human Cell Culture
Source: Antioxidants (Basel). 2023 Oct 2;12(10):1824. doi: 10.3390/antiox12101824 (PMC10604116; doi:10.3390/antiox12101824)
Supplement: Supplementary file 1 [file antioxidants-12-01824-s001.zip › antioxidants-2559860-supplementary.pdf]

Supplementary Materials

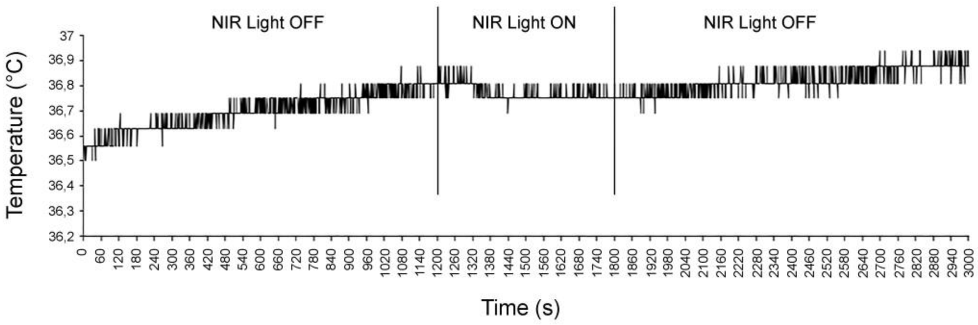

Fig. S1. Measure of real-time cell temperature fluctuation during NIR exposure. Temperature have been measured every second by a waterproof temperature sensor immersed in the cell culture medium. Temperature recording began 20 min before NIR exposure, during the 10 min exposure and continued for 20 min after the end of exposure.

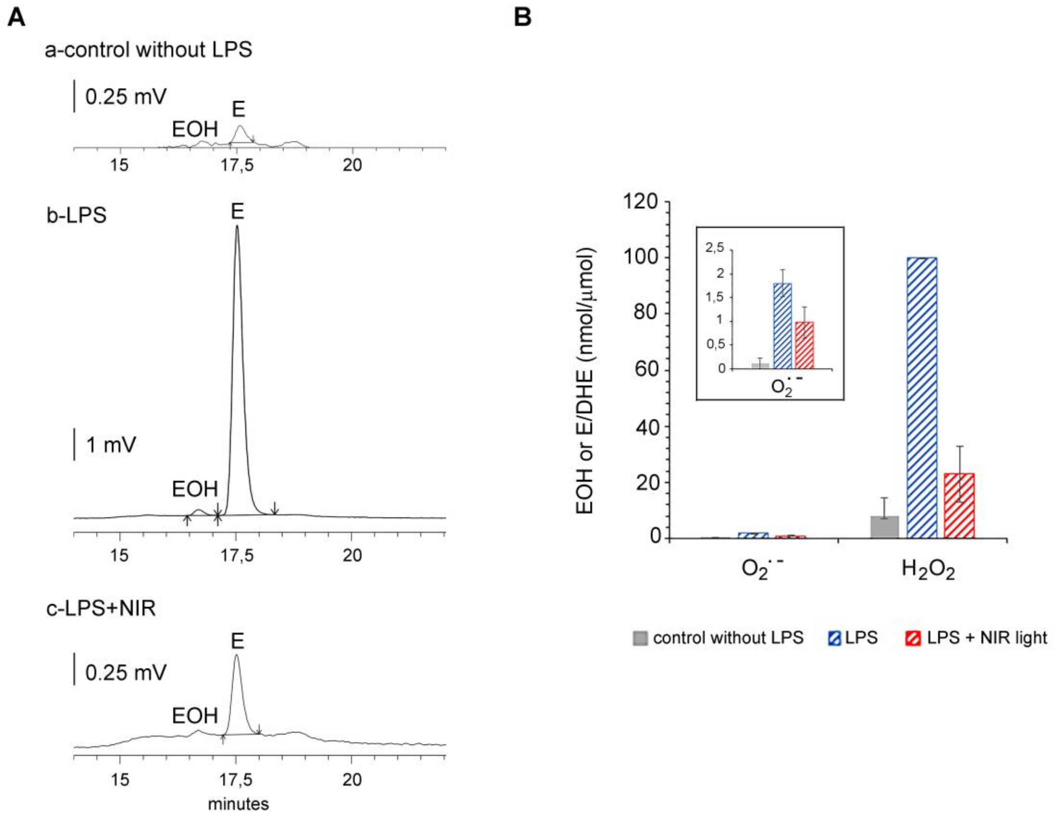

Fig. S2. HPLC analysis of ROS products after NIR light exposure. After activation by LPS for 24h and 10 min exposure to NIR light, HEK-Blue™-hTLR4 cells were maintained in PBS and incubated with 50 µM DHE for 30 min. Cells were then harvested in acetonitrile, dried, resuspended in PBS/DTPA and analyzed by HPLC. (A) representative chromatogram profiles of EOH and E (Ethidium) separation for (A-a) control, (A-b) LPS or (A-c) LPS +NIR light conditions (B) Quantification of EOH/DHE (that reflect $O_2^{\cdot-}$ concentration) and E/DHE (that reflect H_2O_2 concentration) ratio. n=3, *$p < 0.1$; ** $p < 0.01$; *** $p < 0.001$.

Table S1. List of primers used for quantitative real-time PCR.

Species	Genes	Gene accession number	Forward Primer 5'-3'	Reverse Primer 5'-3'
Human	**IL-6**	NM_000600.5	GGCTGCAGGACATGACAACT	ATCTGAGGTGCCCATGCTAC
	**IL-8**	NM_000584.4	CCACCGGAAGGAACCATCTC	GGGGTGGAAAGGTTTGGAGT
	**TNF-α**	NM_000594.4	CAAGGACAGCAGAGGACCAG	TGGCGTCTGAAGGTTGTTTT
	**IFN-α**	NM_024013.3	AGAATCACTCTCTATCTGAAAGAGAAG	TCATGATTTCTGCTCTGACAACCT
	**IFN-β**	NM_002176.4	CGCCGCATTGACCATCTA	GACATTAGCCAGGAGGTTCT
	**IFN-γ**	NM_000619.3	CTAATTATTCGGTAACTGACTTGA	ACAGTTCAGCCATCACTTGGA
	**IL-10**	NM_000572.3	CGCTGTCATCGATTTCTTCCCT	AGGCATTCTTCACCTGCTCCAC
	**TGF-β**	NM_000660.7	ACTATTGCTTCAGCTCCACGGA	AGTCAATGTACAGCTGCCGCA
	**pro-IL-1β**	NM_000576.3	CCTGTGGCCTTGGGCCTCAA	GGTGCTGATGTACCAGTTGGG
	**CAT**	NM_001752.4	ACCCTCGTGGGTTTGCAGTGA	CGAGCACGGTAGGGACAGTTCA
	**GPX1**	NM_000581.4	TGGGCATCAGGAGAACGCCA	GGGGTCGGTCATAAGCGCGG
	**GPX3**	NM_002084.5	CTGACGGGCCAGTACATTGA	TCCACCTGGTCGGACATACT
	**GSR**	NM_000637.5	AGGAGCTGGAGAACGCTGGC	CAATGGCCCAGAGCAGGCA
	**GAPDH**	NM_002046.7	TGCACCACCAACTGCTTAGC	GGCATGGACTGTGGTCATGAG
